# Consumer Perspectives on Maternal and Infant Health Apps: Qualitative Content Analysis

**DOI:** 10.2196/27403

**Published:** 2021-09-01

**Authors:** Rizwana Biviji, Karmen S Williams, Joshua R Vest, Brian E Dixon, Theresa Cullen, Christopher A Harle

**Affiliations:** 1 Science of Healthcare Delivery College of Health Solutions Arizona State University Phoenix, AZ United States; 2 Graduate School of Public Health and Health Policy City University of New York New York, NY United States; 3 Department of Health Policy and Management Richard M Fairbanks School of Public Health Indiana University Indianapolis, IN United States; 4 Center for Biomedical Informatics Regenstrief Institute Indianapolis, IN United States; 5 Department of Epidemiology Richard M. Fairbanks School of Public Health, Indiana University Indianapolis, IN United States; 6 Department of Family Medicine Indiana University School of Medicine Indianapolis, IN United States; 7 Department of Health Outcomes and Biomedical Informatics University of Florida Gainesville, FL United States

**Keywords:** mHealth, mobile applications, maternal and infant health, smartphones, mobile phone

## Abstract

**Background:**

Despite the popularity of maternal and infant health mobile apps, ongoing consumer engagement and sustained app use remain barriers. Few studies have examined user experiences or perceived benefits of maternal and infant health app use from consumer perspectives.

**Objective:**

This study aims to assess users’ self-reported experiences with maternal and infant health apps, perceived benefits, and general feedback by analyzing publicly available user reviews on two popular app stores—Apple App Store and Google Play Store.

**Methods:**

We conducted a qualitative assessment of publicly available user reviews (N=2422) sampled from 75 maternal and infant health apps designed to provide health education or decision-making support to pregnant women or parents and caregivers of infants. The reviews were coded and analyzed using a general inductive qualitative content analysis approach.

**Results:**

The three major themes included the following: app functionality, where users discussed app features and functions; technical aspects, where users talked about technology-based aspects of an app; and app content, where users specifically focused on the app content and the information it provides. The six minor themes included the following: patterns of use, where users highlighted the frequency and type of use; social support, where users talked about receiving social support from friends, family and community of other users; app cost, where users talked about the cost of an app within the context of being cost-effective or a potential waste of money; app comparisons, where users compared one app with others available in app stores; assistance in health care, where users specifically highlighted the role of an app in offering clinical assistance; and customer care support, where users specifically talked about their interaction with the app customer care support team.

**Conclusions:**

Users generally tend to value apps that are of low cost and preferably free, with high-quality content, superior features, enhanced technical aspects, and user-friendly interfaces. Users also find app developer responsiveness to be integral, as it offers them an opportunity to engage in the app development and delivery process. These findings may be beneficial for app developers in designing better apps, as no best practice guidelines currently exist for the app environment.

## Introduction

### Background

Globally, there is a growing demand for, and use of, mobile smartphone apps to disseminate maternal and infant health information and self-management tools effectively to pregnant and postpartum women [1-5]. This increase in acceptance of digital technologies is attributed to the fact that women find it extremely convenient to seek answers to their questions, with easy access to health-related information throughout the day [6]. Furthermore, these technologies may provide important social support, especially when pregnant women feel isolated, time constrained, or need reassurance [6], and apps have shown the potential to produce positive health behavior changes [7]. Maternal and infant health apps frequently appear on the Apple App and Google Play stores’ list of most downloaded apps, and some have been downloaded over 5 million times [8].

Although popular, consumers often rate maternal and infant health apps lower (ie, fewer star ratings) than other categories of apps [9]. Moreover, the percentage of users who discontinue (ie, uninstall) the use of health-focused apps in general approaches 50% [10]. This disconnect between popularity, ratings, and use may be driven by several factors. One reason could be price—maternal and infant health apps are priced higher than apps targeting other populations and health conditions [9]. Alternatively, app efficacy may be a challenge. Apps with unclear or inaccurate consumer decision support could lead to poor end user choices and potentially undesirable outcomes [11]. Most maternal and infant health-focused apps are developed by non–health care organizations [12]. Furthermore, popular maternal and infant health apps typically include only a minority of behavior change techniques that are found to be useful in health promotion [13]. Apps often fail to provide sources of their information and lack warnings pertaining to the use of this information [5], or the reasons could be related to design and usability. Overall, end users have discontinued apps because of the time-consuming data entry process, hidden costs, use difficulty, lack of data privacy, and lack of maintained interest [10]. User commentaries from women’s health apps indicate that women are in favor of apps that are easy to use, contain new information, and are motivational, whereas areas that need improvements are associated with the quality of graphics, download speed, compatibility with other devices, ability to transfer data onto newer versions, and certification or affiliation with credible organizations [9].

### Objectives

Mobile technology can be an effective platform for delivering resources and interventions, but consumer engagement remains a barrier for uptake and continued use [14]. Therefore, it is imperative to consider the consumer’s perspective on these apps to improve the utility of such resources [14]. The objective of this study is to assess users’ self-reported experiences with maternal and infant health apps, perceived benefits, and general feedback by analyzing publicly available user reviews on two popular app stores—Apple App Store and Google Play Store.

## Methods

### Study Design

We conducted a qualitative assessment of publicly available user reviews from a sample of maternal and infant health apps available in the Apple App Store and Google Play Store. We followed a four-step process to create a data set of user reviews from a set of maternal and infant health apps that offer health education or decision-making support to pregnant women or parents or caregivers of infants. First, we populated a database with the set of available maternal and infant health apps by systematically fetching app details from the Apple App Store [15] and Google Play Store [16] using a Java-based scraper program called Node.js (OpenJS Foundation) [17]. Through this automated process, data are retrieved from the web using http in a central database [18]. Scraping programs automate the extraction of information displayed to users in the same way that chart reviewers record details from a medical chart. Second, we identified an inclusive list of relevant keywords that users might search for when locating apps related to maternal and infant health. Third, we scraped the two app stores for candidate apps and merged and deduplicated the resultant apps first within stores and then across stores. We then applied inclusion and exclusion criteria to identify apps that were eligible for the study. Inclusion criteria were as follows: (1) app description written in English; (2) target users judged as to-be pregnant women, to-be parents, and other caregivers of infant children as primary users; (3) listed in the medical, health and fitness, books and references, and education categories in the Apple App Store or listed in the medical, health and fitness, books and references, education, and parenting categories in the Google Play Store; and (4) described as intending to provide health education and user decision-making support. Exclusion criteria were as follows: (1) app description written in any language other than English; (2) inadequate or no app description provided; (3) target users judged as health professionals, providers, and students in health professions as primary users; (4) listed in all other categories; (5) described solely for purposes such as gestational age or due date calculators, identifying baby names, and shopping for baby products; and (6) apps meant to be used by members and patients associated with special programs (eg, fitness centers, clubs, or other paid memberships) or health care facilities (eg, a clinic or hospital). Fourth, we sampled a set of user reviews from the candidate maternal and infant health apps. The data reflected the app store content as of February 2018. Steps 1 to 3 are described in detail elsewhere [12]. The Indiana University Institutional Review Board approved this study as nonhuman subjects’ research.

### Search Strategy

**Figure 1:**
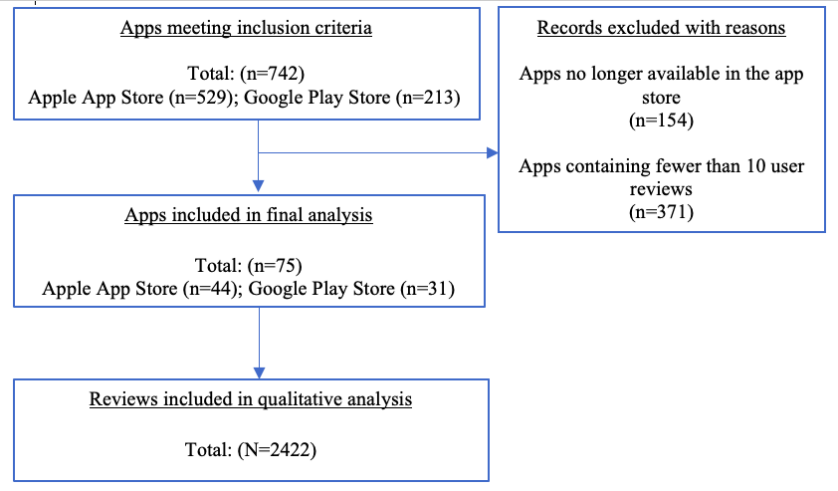
Schematic representation of the user review selection process for qualitative analysis.

The three steps listed earlier resulted in a total of 742 eligible maternal and infant health apps (Figure 1). Of these, 20.8% (154/742) were no longer available in the app stores at the time of data collection (and therefore excluded). Apps with fewer than 10 (371/742, 50%) reviews were also excluded to obtain a robust set of reviews for analyses. From the 217 resulting apps, a simple random sample of 75 (34.6%; 44 Apple App Store and 31 Google Play Store) apps were selected. The sample size was sufficient to achieve data saturation. Each app name was entered into an individual scraper search. Each scrape resulted in maximum allowed user reviews of 50 from the Apple App Store and 40 user reviews from the Google Play Store (ordered by the most recent review). The upper limit per app was set at 40 user reviews for a total sample of 2422 user reviews (Figure 1) to maintain consistency across platforms. In addition to the text of the user reviews, the scraper program returned the star rating, price, and review date (see Multimedia Appendix 1 for a list of apps included in this study). Finally, using a general inductive content analysis approach, we qualitatively analyzed user reviews, where the underlying themes were identified from the data.

### Data Analysis

#### Descriptive Summary

Descriptive statistics were used to summarize the app characteristics such as app price, user star ratings, app platform, and review characteristics such as publication date and length of reviews.

#### Qualitative Analysis

User reviews were analyzed using a general inductive content analysis approach [19,20]. In this approach, themes were derived from data, as opposed to using preconceived categories [21]. First, 2 coders (RB and KSW) undertook a joint reading of a random sample of 30 user reviews to establish consistency in the textual unit of analysis, identification of categories, and formation of themes [20]. Next, the same 2 coders independently read and identified the preliminary codes from a new sample of 200 user reviews. Through joint reading sessions, these preliminary codes were refined, collapsed, assigned descriptive labels, and arranged into a coding framework to be applied to the sample. To assess coding consistency, both the coders independently analyzed a new sample of 100 user reviews. Agreement between the coders was high (κ=0.89). The 2 coders independently coded the remaining sample and met regularly to resolve any coding discrepancies and discuss the themes that were detected in the data. All data management and analyses were conducted in Dedoose 8.0.39 (SocioCultural Research Consultant, LLC) [22].

## Results

### Description of Apps and Reviews

**Figure 2:**
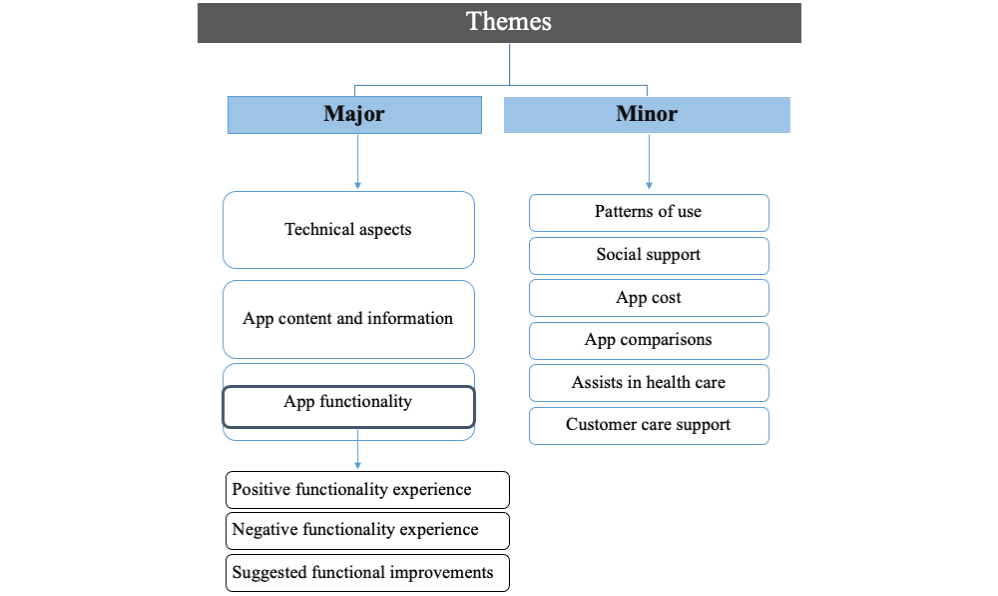
Summary of themes identified from qualitative content analysis.

Of the apps that were included, 75% (56/75) were free. Of the paid apps, the prices ranged from US $0.99 to US $8.99, with an average price of US $3.14 and a median of US $2.99. These prices reflected one-time payments to download the app, not monthly or annual subscription fees. More than half (44/75, 59%) of the apps were from the Apple App Store, and the remaining were from the Google Play Store. The average star rating for all apps was 4.1, with a range of 2.5-5.0. The oldest review in the data was written on July 5, 2009, whereas the latest review was published on February 13, 2018. The longest user review was 403 words long, and the shortest was only 1 word. Three major and six minor themes (present in less than 10% of the reviews) were identified in the data (Figure 2). The definitions of the themes, along with the frequency of occurrence, are presented in Table 1.

**Table 1:** Prevalence of major and minor themes identified in the data (N=2422).

Themes^a^			Definition		Reviews, n (%)
**Major**					
	App functionality	The user specifically talks about app functions and features and what it does either positively or negatively.		2119 (87.49)	
	Technical aspects	The user specifically talks about aspects pertaining to how an app operates either positively or negatively, that is, privacy, security of data, or other technology-based aspects.		510 (21.05)	
	App content	The user specifically talks about the app content and information it provides.		349 (14.41)	
**Minor**					
	Patterns of use	The user highlights the frequency and type of use—whether the app is used for the first pregnancy or the app is being used for a long time.		219 (9.04)	
	Social support	The user specifically talks about receiving support from friends and family or offering support to other women while using the app.		207 (8.54)	
	App cost	The user specifically talks about the cost of an app, ie, cost-effective, or a waste of money.		201 (8.3)	
	App comparisons	The user compares the app with other apps in the market.		104 (4.29)	
	Assists in health care	The user specifically highlights the role of the app in offering clinical assistance.		77 (3.18)	
	Customer care support	The user specifically talks about their interaction with the app customer care support either positively or negatively.		44 (1.82)	

^a^These themes were not exclusive.

### Major Themes

#### App Functionality

Around 87.49% (2119/2422) of user reviews focused on commenting on the functionality of apps, such as app features, indicating that users frequently discuss the functionality of an app while using them. The discussion about app functionality featured three main subthemes: (1) positive functionality experience, (2) negative functionality experience, and (3) suggested functional improvements.

#### Positive Functionality Experience

A majority of the reviews focusing on app functionality indicated an overall positive experience with the functionality of the app. Many of the positive reviews were geared toward an app’s overall design and app features describing these apps as useful, helpful, and easy to use. Users mainly elaborated on app features that assisted in data recording and tracking of data pertaining to infant needs such as feeds, milk pumps, diaper changes, and sleep, akin to a *one-stop shop.* Some of the other tracking features focused on recording the height and weight of the infant, monitoring the trends over time, and keeping a log of immunization and other health histories:

Best tracking all app ever! ibabylog is the best app I have! It helps me track everything from nap times, to meal times, to dirty diapers. Its best feature is that it helps you keep intact with breast feedings. You can track everything in between like medication, doctor visits, growth, anything you can think of. I love this app and I recommend it big time!

Users have also expressed a general preference toward the feature of data sharing and synchronizing of data between two or more devices:

Awesome App! My wife has been using MammBaby on her iPhone and now I am using it on my Android phone. It is definitely the easiest one to use and sharing feature is awesome!

Some of the other features that have received favorable attention include baby heartbeat self-reported monitors, contraction timers, week-by-week fetal development stages, and diet or exercise tips and recommendations:

With countless pregnancy and newborn resources on the market, it can be hard for parents to know where to turn for information. This app is created by experts, for parents. As a mother, I appreciate the easy-to-understand and reassuring information found in the app. Like most pregnancy apps, the week-by-week updates are perfect. And the contraction timer came in handy on the big day! Thank you, Lamaze, for cutting the fuss and giving parents what we really need: power to make the best decisions for our family!

#### Negative Functionality Experience

A frequent complaint pertained to app features that did not meet user expectations. Reviewers expressed concerns over apps that presented inaccurate calculations to users, that is, wrong due dates or height, weight, or percentile calculations:

Inaccurate Percentile Calculation, I don’t know how they’re trying to calculate the percentiles by age, but it’s clearly not working. None of the percentiles in the app have matched up with either the hospital’s or the pediatrician’s data for my son--by a long shot! Even without the doctor’s records telling me he’s somewhere near the 75th percentile, it’s obvious that an 8 lbs. boy is NOT in the 30th percentile for weight for his age at 13 days old, as the app reports.

Users also highlighted issues pertaining to loss of data or inability to synchronize data:

Have to leave app running or else you lose your contractions data. Isn’t that kinda the point in having a contraction app? I will be deleting this one and finding something that actually will track my contractions.

Similarly, there were complaints about apps with no updates or poor updates:

Terrible update! Before this last update on 7/30, the app was fantastic! But now I HATE IT! It’s too cutesy and it’s hard to tell anything anymore! The photo section is far too large now when that is not the information that I need. The daily schedule section is MUCH more confusing, and I can’t see any patterns. I will be deleting and finding a new app now! It is so confusing now. They went with trying to make it look cute instead of functional. Horrible app!!!!

Finally, users reported that they discontinued the use of an app with inadequate features, with limited ability to track data, modify an inaccurate entry, or lacking flexibility in using conversion metrics, that is, mg to oz or kg to lbs:

I deleted it after one time, this app is terrible. It just tells you how much weight you should gain. It doesn’t track your weight or monitor your progress. It doesn’t even save any information. You can find this same information on several sites or easily figure it out on your own. And either way, if you want to keep track, you would have to do it yourself anyway. I deleted the app after the first time I tried it and realized it was useless.

#### Suggested Functional Improvements

Along with critiquing an app’s functionality and features, users often provided additional recommendations pertaining to features that could be added to improve their overall experience with the app. Reviewers often had a bucket list of requests to customize an app and tailor it to their specific needs and requirements. Textbox 1 provides a summary of suggestions for functional improvements. Some of the more common recommendations focused on data visualization or trends data (for height, weight, feedings, or immunizations), ability to export or print data, additional tracking facilities, or capabilities to synchronize data between two or more devices.

**Suggested functional improvements for maternal and infant health apps.**

**Requested Features and Direct Quotes**

- Export data
  “Helpful, but...I would love to be able to export or print data. Otherwise helps me keep track of my preemie between Dr visits.”
- Data visualization and trends data
  “The only thing that would make it better in my opinion would be to have a graph of each activity to show any types of trends. Knowing that my baby has slept 14% more in this 7-day period vs the last 7 days is maybe interesting, but not helpful when I’m trying to see any patterns of when he’s sleeping and for how long. Having a graph that shows when he sleeps each day for a week would be so helpful for getting into more of a natural routine with the baby.”
- Additional data tracking: symptoms, weight, illness, pumping, feeding, and medications
  “Wish it had a medicine tracker and reminder, Wish the app helped with keeping track of when a dose of the medication was given and help with reminders for the next dose.”
- Alerts, alarms, and reminders
  “A Great App!! I use this app all the time! I pretty much rely on it! The only thing I’ve noticed that I’d like them to add is an alarm feature for medicines.”
- Data synchronization between devices and data integration across apps
  “I wish they could allow for other apps to feed info into it. For example, I use a digital thermometer that connects to my phone. If it could sync with this app it would be perfect!”
- Edit or delete incorrect data entries
  “I wish I could edit time entries in case I look to see which side but forgot to click a new entry. But overall, great and easy to use.”

#### Technical Aspects

The second most common theme (510/2422, 21.05%) that users discussed in their reviews were associated with the technical aspects of an app, such as how an app operates. Users preferred apps with a user-friendly app interface, free from advertisements or forced ratings, and those that did not occupy a large space in the phone memory.

However, most of the comments were geared toward technology failures, such as apps that crashed or froze on users, thereby rendering them useless for further use:

Unfortunately, it crashes frequently (usually in the middle of the night when I’m trying to time nursing sessions) and multiples times in a row at that (I think my record is 7). Incredibly irritating when you’re sleep deprived, it’s 2 am, and all you want to do is get back to sleep not continuously open an app all, so it can hang for 30 seconds then crash.

Users have also criticized certain apps that were extremely slow to load the information or content or stream videos:

Generally good, a little slow, switched to this app once the written log from our lactation consultant ran out of space. It’s been very helpful for tracking feedings and diapers. I find, however, that it runs really slowly sometimes in terms of entering information and the nursing/pumping timer.

Other reasons cited as causes for discontinuing app use were inability to register oneself, not available offline, and other glitches associated with synchronizing data or loss of data:

It no longer lets me register or log in, and every time I open it I get the message that its opening for the first time, with a long wait. I probably won’t use it when I’m in labor because of this.

A few users also recommended certain technical improvements that would help enhance their overall experience with the app. These recommendations pertained to app availability on multiple devices and app platforms, or making an app esthetically pleasing (improved user interface, fonts, or image quality):

LOVE, I absolutely love this app, I just wish they would make it Apple Watch compatible as well!

#### App Content and Information

Reviews related to app content or information typically referred to the *wealth of information* these apps had to offer. Users appreciated apps providing detailed information, daily or weekly tips and reminders, latest and updated content, and evidence-based articles:

An evidence-based app you can trust, the information provided by Lamaze comes from evidence-based research, so you know you’re not getting the “fluff” that you read in other pregnancy or parenting apps.

**Figure 3:**
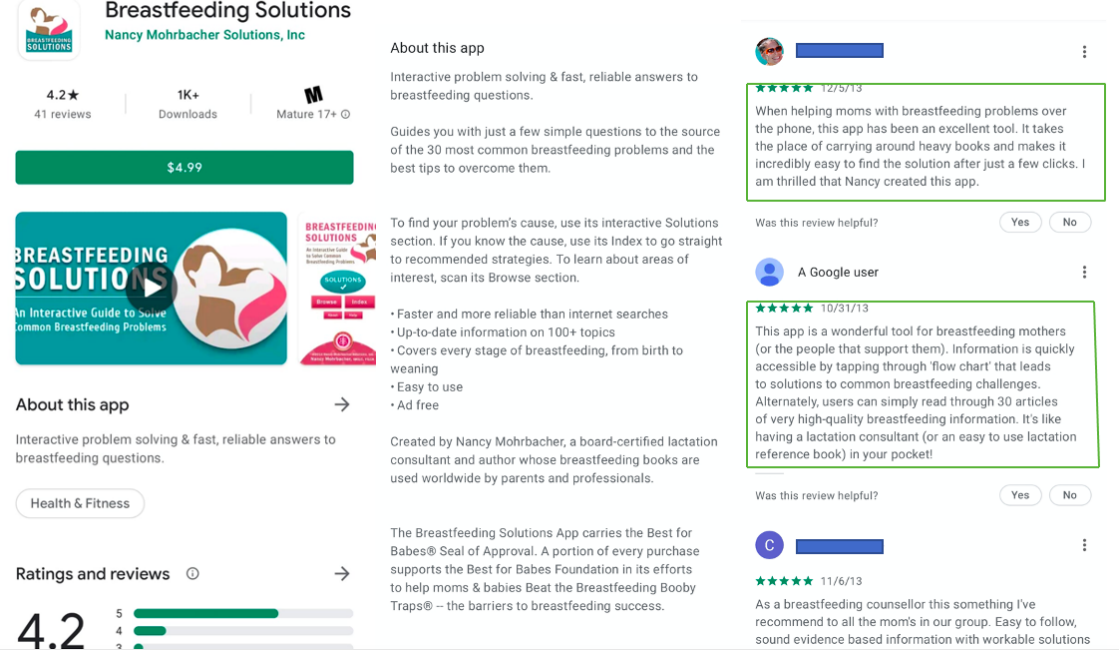
User reviews centered around app content and information.

New parents expressed their satisfaction with apps that met their increased information needs. A few users also suggested that certain apps were better organized and offered greater support as opposed to seeking information on the internet. Other users indicated greater satisfaction with using an app for information seeking over the traditional use of books (Figure 3):

Yes! Love love love this app, used it for my first pregnancy and it put me at such ease being a first time mom, I love how they have something to say everyday because you get so anxious being pregnant and just want the baby and this app helps you calm down and understand every stage. I love that you can put pictures at the bottom and see your progress before the birth of your child.

However, not all users found the content of these apps to be beneficial. Some users articulated a general dislike toward apps that had very brief content, contained basic information that could be found on the internet, or comprised a number of typos or grammatical errors, making it incomprehensible. This was especially true for paid apps, where users had higher expectations in terms of content quality:

Underwhelming application., I cannot speak to the integrity of the information this application gives you, A friend of mine told me to purchase it as I was expecting. For the price I expected much more. The Videos section simply opens their YouTube page in a web browser. And the very limited information the application gives you could fit on one sheet of paper. I was expecting for suggestions on how to deal with or enrich my child’s experience during her leaps, but instead I only found a very brief description of what was happening developmentally. Certain aspects of the application just don’t work at all, you can tell this application was outsourced to international developers for whom English is a second language. I would perhaps give this application three stars if it were free, but considering I paid for it I was expecting more. I’m sure all the information in this app can be found online with little effort. Save your money on this one.

A few users were also disapproving of apps that contained unrelated information or were very narrow in scope; for example, they covered a selective range of topics that were not applicable to many:

I didn’t put five stars because I disapprove the zodiac stuff, I don’t find it useful, fun, or educational (on the contrary).

Furthermore, there were reviews that offered suggestions to improve the app content. Most of the recommendations were geared toward offering content in a different language and adding additional content:

I’ve got a son with one of the rarest of rare genetic diseases that I thought would be awesome for this app it’s called Spinal Muscular Atrophy Respiratory Distress also known as (Smard 1).

Some recommendations were also for simplifying the information to account for the lay audiences:

Hard to understand, the information is correct. However, it would be extremely helpful to nursing mommies if they understood what the medical mumbo jumbo meant!

### Minor Themes

#### Patterns of Use

Over 9.04% (219/2422) of the reviewers described their patterns of app use by identifying the length, duration, and frequency of use. Some of them were long-term users, using these apps for multiple pregnancies. Users have also highlighted their frequency of use, with some users using these apps almost daily. In addition, most app users tend to be first-time parents who use maternal and infant health apps for information seeking, tracking their pregnancy or infant needs, or connecting with other parents for moral support:

Great app! I don’t normally write reviews for apps but this app is amazing. I am a new mom and I use it throughout the day, every day. I use it to keep track of feeding and sleeping trends.

#### Social Support

Over 8.54% (207/2422) of all reviews referred to apps that acted as a safe haven for users, as these apps connected pregnant women and mothers to a larger community of app users, thereby offering social support that is typically needed by many during this period. First-time mothers especially found these communities encouraging and useful to lessen their anxieties over issues where they had minimal experience:

Awesome video blogs, never seen video blogs from other moms before and it helped me feel like I wasn’t the only one. Same goes for the forum. Really good to connect to other moms.

However, a few reviewers were not satisfied with their interactions within such communities. A handful of women faced some level of cyberbullying by others in these communities, especially those who had differing perspectives or values pertaining to childcare. Such negative experiences were geared toward the strong opinions, biases, and immaturity of other users.

Users also expressed an overall appreciation for apps that had the capability to synchronize their data with other caregivers, especially their spouses, family members, or significant others. These features helped parents stay abreast with their infants’ activities at all times:

Great app, I didn’t know a lot about pregnancy and it has helped a lot with the different stages. It has even given my wife some comfort because I know things when she asks me questions.

#### App Cost

**Figure 4:**
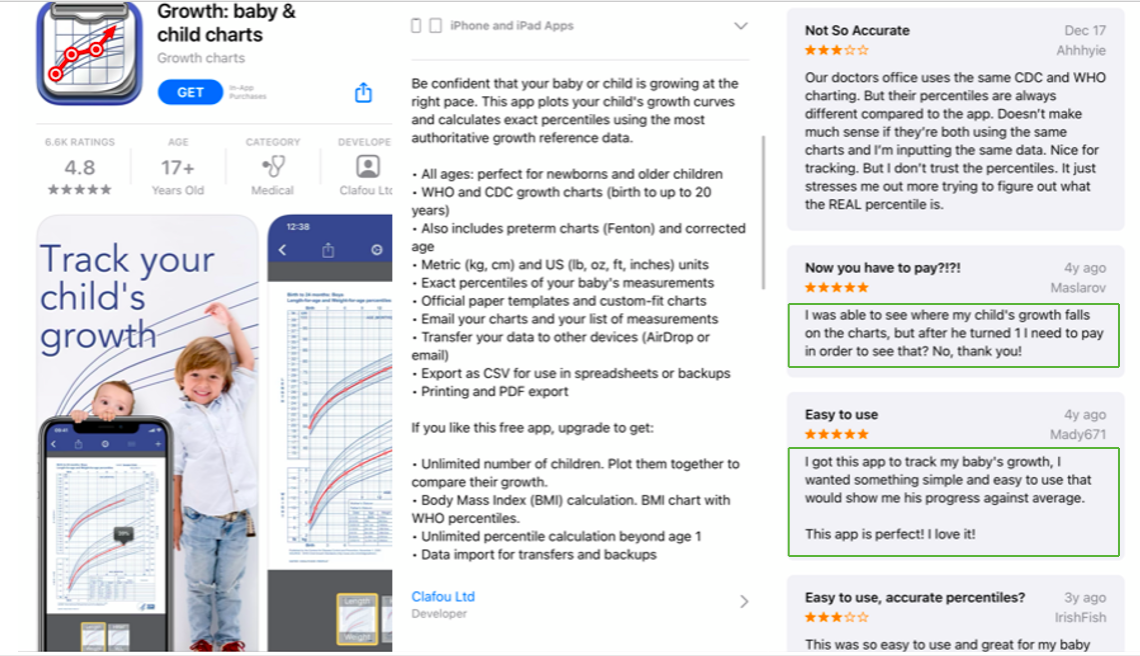
User reviews centered around app cost and ease of use.

Generally speaking, app users preferred apps that were free or had a very low cost associated with it (Figure 4). Users were willing to purchase apps with enhanced capabilities, such as data synchronization on multiple devices, additional tracking features, or data visualization functions. Users were also willing to pay for a *pro* version to eliminate advertisements and pop-ups and appreciated apps that were esthetically appealing.

However, users were disapproving of apps that were labeled as free on the app store but offered very limited functionalities and required an upgrade (pro version) to enjoy additional features. Users called such apps *misleading*, *unfair*, or *false advertising*. Similarly, users were very critical of apps that were paid and did not meet their expectations. Users associated price with quality, thereby voicing extreme distress if the app faced any technical issues, provided basic information, or contained mediocre features:

Lot of glitches, poor info, no matter what article I click on- every other page starts with the same header: “Article good fats from bad ones” and then an incomplete sentence about using safe household cleaners...I tried to leave a review ON one of these pages- because EVERY page asks you for a review, and it said “sorry we couldn’t complete your review right now.” I cannot imagine who would pay 6.99 for this. (Or that they raised the price from 4.99???) I downloaded it for exercises but they don’t have videos to show the exercises so I’m better off just going back to YouTube.

#### App Comparisons

Approximately 4.29% (104/2422) of users compared certain app features, either in a positive or negative context, with other apps that they used in the past or were currently using. Users also compared paid apps with apps that were offered for free to establish whether the app was worth the purchase or not:

Meh, Really...I have a ton of other baby apps that give more details on my baby’s development and what I should be eating. This app is lacking a lot of details I wouldn’t waste my money on going “premium.” I’ll stick with the bump and nurture. Thanks.

#### Assists in Health Care

Approximately 3.18% (77/2422) of app users established the role of maternal and infant health apps in health care management. Consumers especially found these apps useful for keeping track of infants who were underweight, short for age, premature at birth, or with congenital birth defects. They confirmed the role of these apps in recording accurate data that could then be reported effectively to their pediatrician:

I have used this for the last six months with no troubles. It’s quick, intuitive, and easy. I have three kids and this baby was a newborn ICU baby, and this made life simpler with multiple caretakers/doctors who want all the details (and not great communication between all the people involved in baby’s life.) and exhaustion from all the demands. What a lifesaver!

A minority of reviews were initiated by the health care professional, where they specified either using these apps for information seeking or monitoring patients’ progress. Health care professionals have also provided recommendations for certain apps to their patients.

#### Customer Care Support

Users particularly cared about customer care responsiveness in addressing their questions, promptness in fixing issues, as well as fulfilling their requests for additional features. Consumers expressed distress over situations where they paid for an app but did not receive adequate developer support:

Doesn’t work at all, I purchased this app and was so excited after reading the reviews. However, this app is crap! Took my money but does not work at all, nothing. Stays on one page and that’s it. I can’t get in touch with app support either. Don’t waste $$$ or time. I have tried reinstalling it several times and still crap.

## Discussion

### Principal Findings

To our knowledge, this is the first study to evaluate consumer preferences for mobile apps targeted toward maternal and infant health using publicly available user review data. The elements of maternal and infant health apps that users were most interested in were related to the functionality of apps, that is, app features. Users were satisfied with apps that offered advanced features such as data monitoring and tracking or data synchronizing abilities across different devices. Similarly, users were highly critical of apps that did not meet user expectations in terms of their functionality and were prompted to discontinue the use of apps with limited functionalities. A large number of comments were concerned with the loss of data or inability to edit an incorrect entry. The overall emphasis on app functionality is consistent with previous literature that highlights the role of user satisfaction with app functions as one of the major caveats in consumer app use [14,23]. In addition, users also offered suggestions or recommendations in terms of app features that would improve their overall experience with the app. This is again consistent with previous reports in other domains [14,24,25], indicating that consumers are not fully content with the available features and their needs are often inadequately addressed.

Consumers prefer apps that are easy to use, with an esthetically pleasing interface, and occupy less memory space. The reasons cited for discontinuation of app use centered around technical issues such as crashing or freezing of apps while in use, slow download speed, and too many pop-ups or forced ratings [9,26,27]. An app that crashes often is deleted by users before any engagement with key functions has occurred [23]. Thus, app developers may give careful consideration to the technical aspects of these apps before releasing them for use. One of the major aspects of consideration for app use is the quality of information or content provided by these apps. When it comes to content, users typically value tailored information pertaining to their condition and actionable solutions for its effective management. Pregnant women and new mothers often seek information from apps on topics such as establishing breastfeeding, solving breastfeeding problems, infant health issues, and topics that are uncomfortable to discuss with health care providers [28]. Overall, women reported a positive experience with pregnancy apps, but a few reported issues with what they perceived as validity, accuracy, and timeliness of the information that was being presented by certain apps.

App cost was recognized as an important consideration for app adoption and use. Most users preferred free apps; however, users were willing to pay for apps if they offered sophisticated features and comprehensive information and were of superior quality. These results are consistent with those of previous studies on maternal and infant health apps [12]. Although some users discussed a negative experience with app-based communities, most women expressed their enthusiasm in terms of the social support they received from fellow mothers undergoing similar experiences. Women value peer experiences and knowledge on important topics such as breastfeeding or infant care and, in turn, offer similar support to others in need. Women often use these platforms to discuss topics that are sensitive in nature, such as sexual activity during and after pregnancy or feelings of hopelessness [29]. The anonymity offered in such communities provides an opportunity to raise issues that they otherwise would not discuss with family, friends, or health care professionals during in-person visits [29]. Aside from being a part of an ongoing community of app users, women have also cherished the ability to engage their partners and other family members in maternal and infant health care and support. Information seeking during pregnancy and postpartum was relatively higher among first-time mothers who use these apps to track stages of pregnancy on a weekly basis or monitor infant development. Women also report using these apps for multiple pregnancies, and some engage with maternal and infant health apps almost daily.

Increasingly, women are using maternal and infant health apps to assist them in health literacy, monitoring, self-management, as well as consumer decision-making. Reviewers elucidated the role of maternal and infant health apps in improving patient-provider communication by aiding in data tracking, which helps improve recall and increase preparedness for doctor visits. Certain apps offer additional features where users can print reports or directly email them to their providers. This increased use of mobile apps during pregnancy and postpartum periods also highlights the importance of providing evidence-based information, especially because of the vulnerable nature of these phases. Very few reviewers discussed the availability of evidence-based content or expressed a desire to identify the scientific sources of information that are being presented to them. This may be credited to the fact that users are more concerned with the overall appeal of an app in terms of functionality, features, content, or usability, as opposed to verifying the credibility of this information. Therefore, health care professionals, app developers, and policy makers may consider strategies to review and promote apps to consumers based on information accuracy and trustworthiness. Future research may focus on evaluating the quality of maternal and infant health app content and information.

Furthermore, there is increased satisfaction among users whose needs and viewpoints were adequately addressed by the app developers. This highlights the fact that consumers value increased involvement in the app development and delivery process, which may increase their engagement and long-term use.

These results may provide meaningful information for app developers and other stakeholders regarding consumer needs and expectations pertaining to maternal and infant health apps, which are often complex and multidimensional. App developers may also consider strategies to collaborate with health care professionals for app content development and technical experts for app interface and usability to provide evidence-based information with superior functionality to users at low cost.

### Strengths and Limitations

A major strength of this study is that it analyzes consumer attitudes and perspectives toward maternal and infant health apps using a large and diverse sample of publicly available user review data from Apple App and Google Play stores. This study uses a systematic approach of sampling a comprehensive set of user reviews for analysis. Importantly, to our knowledge, this is the first study that uses this particular methodology and sampling strategy to study users’ perspectives on maternal and infant health apps.

This study had some limitations. First, it is likely that user reviews on the app store may not be representative of the larger population of app users. User opinion on a given app may be affected by gender, economic status, education level, or other demographics. However, these data elements were not publicly available and were not considered to draw conclusions. The study, therefore, uses information similar to what would normally be available to consumers in a *real-world* context before downloading an app. In addition, the study used a random sample of user reviews using a strategic approach to capture a more diverse sample of app users, which probably could not be achieved using other exploratory methods such as interviews or focus groups. Second, our results may not be generalizable to apps belonging to other health domains; for example, app use for monitoring infant needs may not be applicable to other populations such as for diabetes or weight loss. Third, the motivation to post may be to give a negative review to express dissatisfaction with the app [30]. However, in our random sample of apps, positive reviews were more common, which is consistent with previous reports [14,31]. Finally, there is a possibility of fake reviews to increase the app demand [32]; however, the large number of reviews per app should compensate for this challenge.

### Conclusions

This review extends the literature by emphasizing the features of maternal and infant health apps that are particularly important to users. These results may be beneficial for app developers to consider during app development. Overall, consumers value low-cost apps that have high-quality content, superior features, and smooth technical aspects and are easy to use. Users consider app developer responsiveness an integral part of app use, as it empowers them in the process of app development and delivery. These consumer perspectives are essential for mobile health sustenance, as no best practice guidelines currently exist for the app environment. Users are increasingly using apps for health care management and informed decision-making. Thus, health care professionals, app developers, and policy makers may consider strategies to review and promote evidence-based and trustworthy apps to consumers. Future studies may focus on assessing user experiences by using other qualitative methods to garner detailed perspectives on long-term app use. This study should also be replicated in other health domains to gain a greater sense of consumer perspectives in the field of mobile health. Future studies may also focus on developing a framework for consumers to evaluate app quality for effective app comparisons and use decisions.

## Appendix

Multimedia Appendix 1 List of apps included in the qualitative analysis.
