# Supplementary material for: Consumer Perspectives on Maternal and Infant Health Apps: Qualitative Content Analysis
Source: J Med Internet Res. 2021 Sep 1;23(9):e27403. doi: 10.2196/27403 (PMC8444044; doi:10.2196/27403)
Supplement: Multimedia Appendix 1 [file jmir_v23i9e27403_app1.docx]

Appendix 1: List of Apps Included in Qualitative Analysis

| **Platform** | **Title** | **Developer** | **Number of Scraped Reviews** | **Star Ratings** | **Price** |
| --- | --- | --- | --- | --- | --- |
| iOS | Pregnancy & Baby \| What to Expect | Everyday Health, Inc. | 40 | 4.5 | 0 |
| iOS | Baby Nursing - Breastfeeding Tracker | Sevenlogics, Inc. | 40 | 4.5 | 0 |
| iOS | BabyBump Pregnancy Pro with Baby Names | Alt12 Apps, LLC | 40 | 4.5 | 0 |
| iOS | iPregnancy (Pregnancy App) | Gregory P. Moore, MD | 40 | 4 | 3.99 |
| iOS | Pregnancy Countdown – Weekly Fetus & Mother Development plus Tips, Information and Checklists | Pregniful Solutions LTD | 40 | 3 | 0 |
| iOS | Baby Tracker (Feed timer, sleep, diaper log) | Nighp Software LLC | 40 | 4.5 | 0 |
| iOS | MammaBaby - Breastfeeding Logger | Life'n Stats | 40 | 4.5 | 0 |
| iOS | iPregnant Pregnancy Tracker Free (iPeriod's Pregnancy Companion) | Winkpass Creations, Inc. | 40 | 4 | 0 |
| iOS | Growth: charts for baby and child tracking | Clafou Apps | 40 | 4.5 | 0 |
| iOS | The Wonder Weeks | Domus Technica | 40 | 2.5 | 1.99 |
| iOS | Pregnant Dad | SB Apps | 40 | 3.5 | 1.99 |
| iOS | iBabyLog: Baby Breastfeeding Timer, Nursing Tracker and Sleep, Diaper, Activities Log | Palanati Group, LLC | 40 | 4.5 | 0 |
| iOS | Pregnancy Weight Calculator & Baby Bump Weight Gain from Mobile Mom | Rebellion Media | 40 | 3.5 | 0 |
| iOS | Contraction Timer Deluxe | Deltaworks | 34 | 4 | 0.99 |
| iOS | Baby Tracker & Digital Scrapbook \| Kidfolio Pro | Alt12 Apps, LLC | 40 | 4.5 | 0 |
| iOS | BabyTime Baby Feeding Timer - Breastfeeding & More | Enhancient | 40 | 4.5 | 2.99 |
| iOS | Contraction Timer and Fetal Kick Counter | Quality Work Software llc | 10 | 3.5 | 0.99 |
| iOS | Genetics & Birth Defects: Medical Dictionary and Terminology of Human Genetic Code and Evolution | Michael Quach | 10 | 3 | 0 |
| iOS | Hatch Baby - Breastfeeding, Sleep, & Diaper tracker | Hatch Baby, Inc. | 40 | 4.5 | 0 |
| iOS | MyDueDate - pregnancy progress tracker | Aspyre Solutions | 38 | 3 | 0.99 |
| iOS | Milk Maid | Michael Kale | 40 | 4.5 | 2.99 |
| iOS | WomanLog Pregnancy Calendar | Pro Active App | 21 | 4 | 0 |
| iOS | Contraction Timer - Time labor contractions | PENGUIN APPS PTY LTD | 40 | 4.5 | 0 |
| iOS | Pregnancy Pounds - Weight Tracking App | Squallsoft LLC | 16 | 4.5 | 3.99 |
| iOS | Happy Pregnancy Ticker | SOFTCRAFT SYSTEMS AND SOLUTIONS PRIVATE LIMITED | 11 | 4 | 0 |
| iOS | Baby Kicks Monitor - Fetal Movement & Kick Counter | Maxwell Software | 11 | 5 | 0.99 |
| iOS | Positive Pregnancy with Andrew Johnson | Michael Schneider | 30 | 4.5 | 2.99 |
| iOS | Totally Pregnant - A Total Pregnancy Experience | 40weeks | 40 | 3.5 | 0 |
| iOS | Kids' Wellness Tracker | McNeil-PPC, Inc | 40 | 3.5 | 0 |
| iOS | Contraction Timer Lite | Michael Kale | 20 | 4 | 0 |
| iOS | Breastfeeding Central | Andrew Rae | 30 | 4 | 3.99 |
| iOS | Pumping Tracker - Breast Milk Pump Log for Mama | LINKLINKS LTD | 40 | 4 | 0 |
| iOS | Baby Daily Activity Tracker tools iCareRoom Free | Yangwoo Park | 18 | 4 | 0 |
| iOS | Pregnancy — Tracker, Assistant & Calendar | Aliaksei Khanenia | 18 | 5 | 0 |
| iOS | Prenatal Yoga - Pregnancy Fitness | Dawnsun Technologies LLC | 20 | 3 | 0 |
| iOS | Baby Log PRO - Feed Timer Breastfeeding Tracker | ChuChu Train Productions | 12 | 4.5 | 1.99 |
| iOS | Baby Care (Feeding, Sleep and Diaper Track & Log for Newborn) | Yi Ding | 11 | 4 | 2.99 |
| iOS | Baby Kick Counter & Monitor - Fetal movement and pregnancy tracker. | BabymedLLC | 17 | 3.5 | 0 |
| iOS | Which Boob? Simplified breastfeeding tracker for nursing Moms | Christopher Hardy | 20 | 4.5 | 0 |
| iOS | Pregnancy View | Fertility Council | 12 | 4.5 | 0 |
| iOS | Baby2Body. | Baby2Body Limited | 25 | 3 | 0 |
| iOS | Breastfeeding Myths - Guide for Lactation | Jorge Gregorio Martin Bello | 10 | 4.5 | 0 |
| iOS | Expectful | Expectful LLC | 40 | 5 | 0 |
| iOS | Hear My Baby - Baby Heartbeat Monitor App | Fat Cigar Productions Ltd | 37 | 4 | 3.99 |
| Android | Feed Baby - Baby Tracker | Penguin Apps | 40 | 4.5 | 0 |
| Android | BabyBump Pregnancy Pro | Alt12 Apps, Inc. | 40 | 4.5 | 0 |
| Android | I'm Pregnant / Pregnancy App | Pregnancy & Baby App | 40 | 4.4 | 0 |
| Android | Feed Baby Pro - Baby Tracker | Penguin Apps | 40 | 4.6 | 8.99 |
| Android | Pregnancy Calendar and Tracker | Mobile Dimension LLC | 40 | 4.8 | 0 |
| Android | Baby Tracker - Feed,Diaper Log | NIGHP SOFTWARE | 40 | 4.6 | 0 |
| Android | Baby Connect (activity log) | Seacloud Software | 40 | 4.7 | 4.99 |
| Android | Breastfeeding Tracker Pumping | Whisper Arts | 40 | 4.4 | 0 |
| Android | Baby Manager - Breastfeeding | InnMov Software | 40 | 4.7 | 0 |
| Android | First Time Pregnancy | amiiSolutions | 40 | 4.3 | 0 |
| Android | Glow Baby for Breastfeeding | Glow Inc | 40 | 4.6 | 0 |
| Android | Pregnancy Companion by OBGYN | EmbraceHer Innovations, Inc. | 40 | 3.9 | 0 |
| Android | Easy Contraction Timer | Cuberob | 40 | 4.3 | 0 |
| Android | Prenatal Lullabies Lite | IMOBLIFE INC. | 40 | 4.2 | 0 |
| Android | LactMed | National Library of Medicine at NIH | 40 | 4.3 | 0 |
| Android | Babylog - daily/growth tracker | ForestApps | 40 | 4.6 | 0 |
| Android | ANMOL | Ministry of Health & Family Welfare | 40 | 3.7 | 0 |
| Android | YOUR BABY'S HEARTBEAT ANYTIME! | Fetal Beats Inc. | 40 | 2.7 | 0 |
| Android | Prenatal Yoga (PRO) | Daily Yoga Software Technology Co. Ltd | 40 | 3.2 | 0 |
| Android | Full Term - Contraction Timer | Mustansir Golawala | 40 | 4.2 | 0 |
| Android | Pregnancy Workouts by Power 20 | Power 20 | 40 | 4.3 | 2.99 |
| Android | latchME - breastfeed easier | Jonathan Goldfinger | 40 | 3.5 | 0 |
| Android | Moms Into Fitness | Moms Into Fitness, Inc. | 40 | 3.6 | 0 |
| Android | MammaBaby Breastfeeding Logger | MammaBabyAndroid | 38 | 4.5 | 0 |
| Android | Pregnancy to Parenting | Lamaze International | 40 | 3 | 0 |
| Android | MuM | Techtree IT Systems Private Limited | 21 | 4 | 0 |
| Android | See Baby Pregnancy Guide | EHD | 24 | 3.9 | 0 |
| Android | Pregnancy Health & Fitness | SparkPeople | 13 | 3.4 | 0 |
| Android | Breastfeeding Solutions | Nancy Mohrbacher Solutions, Inc | 15 | 4.5 | 4.99 |
| Android | Pregnancy yoga Exercises | Home Fitness | 25 | 3.9 | 0 |
| Android | Contraction timer for labor | Henry Naftulin | 15 | 4.4 | 0 |
